# Editorial: Beyond Space-Based or Feature-Based Selection: Mechanisms of Object-Based Attention

**DOI:** 10.3389/fnint.2016.00039

**Published:** 2016-11-22

**Authors:** Vivian M. Ciaramitaro, Gene R. Stoner

**Affiliations:** ^1^Department of Psychology, University of Massachusetts BostonBoston, MA, USA; ^2^The Vision Center Laboratory, Salk Institute for Biological Studies, Systems NeuroscienceLa Jolla, CA, USA

**Keywords:** space-based attention, feature-based attention, object-based attention

Our senses are constantly bombarded by an overwhelming amount of information, yet, our brain has a limited computational capacity and can fully process only a small fraction of that information at any given time. By selectively attending to information we overcome this limited capacity, prioritizing the processing of behaviorally relevant sensory information to the detriment of behaviorally irrelevant sensory information.

Mechanisms underlying attentional selection and the prioritization of select locations in space or select features, such as visual color or motion, have been investigated in considerable detail (e.g., Carrasco, 2011). Our perceptual experience, however, is not one of disjoint features at disparate spatial locations, but of unified representations, objects, which can serve as the goal of our actions. Relative to our understanding of space- and feature-based selection, much less is known regarding object-based selection (see Chen, 2012 for review). In object-based selection, the object is the unit of selection such that if one feature of an object is attended, other task irrelevant features of that object are also prioritized (e.g., Duncan, 1984; O'Craven et al., 1999). How object-based mechanisms might differ from other forms of feature-based selection is poorly understood. For example, in global feature-based attention, selecting a feature prioritizes processing of this feature across the visual field, irrespective of location (e.g., Boynton et al., 2006; Liu and Mance, 2011; reviewed in Treue and Martinez-Trujillo, 2007).

The definition of a perceptual object has long been the focus of both philosophical and empirical inquiry. Our ability to perceive visual objects is present from early in development (e.g., Spelke, 1990; Leslie et al., 1998 offers a comparison of the object concept in development and object-based attention). Our perception of objects is, in part, based on mechanisms of perceptual grouping, such as Gestalt principles, and attention can be, in turn, influenced by this grouping (e.g., Harms and Bundesen, 1983; Driver and Baylis, 1989; Kramer and Jacobson, 1991; Baylis and Driver, 1992).

One fundamental question is whether or not attention does automatically spread across all features and locations delineated by object boundaries. Given that the spreading of attention across an entire object may not always be optimal and may not be mandatory, another fundamental question is how subsets of an object might be prioritized and enhanced, while task-irrelevant subsets of an object might be suppressed. Whereas, some studies have considered a more “pure” form of object-based selection, trying to control for the confounds of space- or feature-based selection (e.g., Mitchell et al., 2003, 2004; Ciaramitaro et al., 2011), other studies have focused on how space- or feature-based selection are constrained by objecthood (e.g., Shomstein and Yantis, 2004; Shomstein and Behrmann, 2008).

Several of the contributions to our research topic consider the diverse factors that can influence the allocation of attention within and between visual objects. *Spatial uncertainty* can play a role in re-allocating spatial attention within object boundaries. To decouple the influence of space- and object-based attention, Drummond and Shomstein present a cue with either high or low certainty within an object and sample behavior at several time points following cue presentation. They find that as spatial uncertainty decreases, spatial information guides selection and objects are filtered out. *Reward history* can also determine the allocation of attention. Sali et al. find that inconsistent vs. consistent rewarding based on a feature, such as color, can bias which of multiple objects are attended. *Behavioral relevance* also plays a role. Lim and Sinnett study *attention set*, a proxy for behavioral relevance, and find that the influence of a peripheral cue on a central task varies depending on whether peripheral cues and central targets contain objects from the same category or shared features. *Hierarchical internal representation* of a visual object may also influence how attention is allocated to an object. Valdés-Sosa et al. find that attention may act at different levels of the object hierarchy and that spatial frequency information structures the organization of object hierarchy. Another key element is that of *perceptual grouping*. Freeman et al. focus on the fate of task-irrelevant features when perceptually grouped as belonging to an object vs. ungrouped. They find that neuronal mechanisms of object-based attention, as assessed by fMRI, are not purely facilitatory and do not automatically spread across hemifields for objects delineated via perceptual grouping. They argue that suppression of task-irrelevant stimuli may depend on how effectively they compete with task-relevant stimuli, with greater competition when relevant and irrelevant features or locations belong to the same object.

Two other contributions consider models that allow for the selection of behaviorally relevant visual objects amongst a crowd of irrelevant objects, exploring the influence of emotion and categorization (Chang et al.), and delineating a new model of visual crowding that can account for object-level crowding (Chaney et al.).

*Sensory modality* is another key dimension in object-based attention. While the majority of contributions to our research topic focus on visual objects, perceptual objects are also present in other sensory domains, such as audition (e.g., Griffiths and Warren, 2004; Bizley and Cohen, 2013). Much less is understood about the processing of such non-visual objects. Bharadwaj et al. highlight that whereas the visual steady-state response is clearly modulated by visual attention, studies examining the auditory analog, the auditory steady-state response, have found inconsistent effects from auditory attention. Using naturalistic auditory objects and methodological advances, they resolve some of the inconsistencies from previous findings and provide evidence that the two forms of selection may act similarly. Specifically, they find that the auditory steady-state brain response can be selectively enhanced for attended objects and suppressed for unattended objects as has been found for visual objects and the visual steady-state response.

Finally, the origins and development of different mechanisms of attention are still poorly understood. One contribution to our research topic addresses how *emotional or social salience* can guide attentional selection, considering such influences early in development, not simply in adulthood. Valenza et al. examine this influence for the unique case of faces. They find differences in adults vs. infants, suggesting that experience with select objects may influence object-based selection.

To conclude, the diverse contributions offered here reveal the multi-faceted nature of attending within and between objects and point the way to further explorations of those facets, especially in domains where our understanding is more limited, such as considerations of objects in other sensory domains and the development of many of these select mechanisms of attention.

## Author contributions

All authors listed, have made substantial, direct and intellectual contribution to the work, and approved it for publication.

### Conflict of interest statement

The authors declare that the research was conducted in the absence of any commercial or financial relationships that could be construed as a potential conflict of interest.

## References

[B1] Baylis G. C. Driver J. (1992). Visual parsing and response competition: the effect of grouping factors. Percept. Psychophys. 51, 145–162. 10.3758/BF03212239 1549433

[B2] Bizley J. K. Cohen Y. E. (2013). The what, where and how of auditory-object perception. Nat. Rev. Neurosci. 14, 693–707. 10.1038/nrn3565 24052177 PMC4082027

[B3] Boynton G. M. Ciaramitaro V. M. Arman A. C. (2006). Effects of feature-based attention on the motion aftereffect at remote locations. Vision Res. 46, 2968–2976. 10.1016/j.visres.2006.03.003 16698060

[B4] Carrasco M. (2011). Visual attention: the past 25 years. Vision Res. 13, 1484–1525. 10.1016/j.visres.2011.04.012 PMC3390154 21549742

[B5] Chen Z. (2012). Object-based attention: a tutorial review. Atten. Percept. Psychophys. 74, 784–802. 10.3758/s13414-012-0322-z 22673856

[B6] Ciaramitaro V. M. Mitchell J. F. Stoner G. R. Reynolds J. H. Boynton G. M. (2011). Object-based attention to one of two superimposed surfaces alters responses in human early visual cortex. J. Neurophysiol. 105, 1258–1265. 10.1152/jn.00680.2010 21228306 PMC3074415

[B7] Driver J. Baylis (1989). Movement and visual attention: the spotlight metaphor breaks down. J. Exp. Psychol. Hum. Percept. 15, 448–456. 10.1037/0096-1523.15.3.448 2527954

[B8] Duncan J. (1984). Selective attention and the organization of visual information. J. Exp. Psychol. Gen. 113, 501–517. 10.1037/0096-3445.113.4.501 6240521

[B9] Griffiths T. D. Warren J. D. (2004). What is an auditory object? Nat. Rev. Neurosci. 5, 887–892. 10.1038/nrn1538 15496866

[B10] Harms L. Bundesen C. (1983). Color segregation and selective attention in a nonsearch task. Percept. Psychophys. 33, 11–19. 10.3758/BF03205861 6844088

[B11] Kramer A. F. Jacobson A. (1991). Perceptual organization and focused attention: the role of proximity in visual processing. Percept. Psychophys. 50, 267–284. 10.3758/BF03206750 1754368

[B12] Leslie A. Xu F. Tremoulet P. D. Scholl B. J. (1998). Indexing and the object concept: developming ‘what’ and ‘where’ systems. Trends Cogn. Sci. 2, 10–18. 10.1016/S1364-6613(97)01113-3 21244957

[B13] Liu T. Mance I. (2011). Constant spread of feature-based attention across the visual field. Vision Res. 51, 26–33. 10.1016/j.visres.2010.09.023 20887745

[B14] Mitchell J. F. Stoner G. R. Fallah M. Reynolds J. H. (2003). Attentional selection of superimposed surfaces cannot be explained by modulation of the gain of color channels. Vision Res. 43, 1323–1328. 10.1016/S0042-6989(03)00123-8 12742102

[B15] Mitchell J. F. Stoner G. R. Reynolds J. H. (2004). Object-based attention determines dominance in binocular rivalry. Nature 429, 410–413. 10.1038/nature02584 15164062

[B16] O'Craven K. M. Downing P. E. Kanwisher N. (1999). fMRI evidence for objects as the units of attentional selection. Nature 401, 584–587. 10.1038/44134 10524624

[B17] Shomstein S. Behrmann M. (2008). Object-based attention: strength of object representation and attentional guidance. Percept. Psychophys. 70, 132–144. 10.3758/PP.70.1.132 18306967 PMC2739629

[B18] Shomstein S. Yantis S. (2004). Configural and contextual prioritization in object-based attention. Psychon. Bull. Rev. 11, 247–253. 10.3758/BF03196566 15260189

[B19] Spelke E. S. (1990). Principles of object perception. Cogn. Sci. 14, 29–56. 10.1207/s15516709cog1401_3

[B20] Treue S. Martinez-Trujillo J. C. (2007). Attending to features inside and outside the spotlight of attention. Neuron 55, 174–176. 10.1016/j.neuron.2007.07.005 17640519

